# Curved Film Microstructure Arrays Fabricated via Mechanical Stretching

**DOI:** 10.3390/mi12111281

**Published:** 2021-10-20

**Authors:** Qiushu Zhang, Bei Peng, Mengqi Chu, Pan Wen, Song Wang, Jintao Xu

**Affiliations:** School of Mechanical and Electrical Engineering, University of Electronic Science and Technology of China, Chengdu 611731, China; qiushuzhang@uestc.edu.cn (Q.Z.); mengqi.chu@unisoc.com (M.C.); 201922040513@std.uestc.edu.cn (P.W.); wangsong-b7@boe.com.cn (S.W.); xujintao@eswin.com (J.X.)

**Keywords:** microstructure arrays, film buckling, mechanical stretching, PDMS

## Abstract

We report on curved film microstructure arrays fabricated through polydimethylsiloxane (PDMS) film buckling induced by mechanical stretching. In the process of the microstructure preparation, a PDMA film is glued on a bidirectionally prestretched PDMS sheet that has a square distributed hole array on its surface. After releasing the prestrain, the film microstructure array is created spontaneously. The fabricated microstructures possess a spherical surface and demonstrate very good uniformity. The film microstructure arrays can serve as microlens arrays with a focal length of 1010 μm. The microstructure formation mechanism is investigated via theoretical analysis and numerical simulation. The simulation results agree well with the experimental results. The prestrain applied by mechanical stretching during the fabrication has an important effect on the shape of the resulting film microstructures. The microstructure geometry can be easily tuned through controlling the applied prestrain.

## 1. Introduction

Polydimethylsiloxane (PDMS) is an elastomer with many interesting properties. It has high transparency at wavelengths from UV to the visible regions (300–800 nm). It is chemically inert, biocompatible, thermally stable, nontoxic, and commercially available. Therefore, PDMS has attracted much attention for different applications in different fields such as optics [1,2], electronics [3,4], and soft robotics [5,6,7,8]. Additionally, PDMS can also be used to fabricate microfluidic devices [9,10]. However, PDMS-based microfluidic devices are not suited to mass production. Some other polymers are potential alternatives to PDMS, which include polymethyl methacrylate (PMMA), polystyrene (PS), polypropylene (PP), cyclo-olefin polymer (COP), and cyclic olefin copolymer (COC) [11,12].

Thanks to its excellent contour accuracy (<10 nm), PDMS has been widely used in the micro- and nanotechnologies [13]. The generation of PDMS surface microstructures or nanostructures is of great significance for various applications that include optical elements [14,15], bioinspired robots [6], control of friction or adhesion [16,17], and so on. The PDMS surface patterns can be achieved by molding/casting and/or soft lithography with high fidelity [10,14,18,19,20,21]. For example, Cadarso, et al. reported a three-dimensional (3D) modulable PDMS-based microlens system that was fabricated through replicating the pattern on silicon [21]. The fabrication started with the definition of the microstructure on silicon, from which the SU-8 master was obtained. Finally, PDMS was cast over the SU-8 master to form the microlens system. Wrinkling from bilayer thin films are also utilized to create PDMS surface patterns, which can be induced by heating, solvent swelling, mechanical stretching/compression, etc. [22,23,24,25,26,27]. As an example, Ma et al. fabricated a PDMS/Au grating by means of surface wrinkling [25]. The manufacturing process involves three stages: applying uniaxial prestrain to a PDMS slab by elastically stretching; sputter-coating the prestrained PDMS slab with a gold film of nanoscale thickness; and, finally, releasing the prestrain to form the wrinkles in both the gold film and PDMS substrate surface in a sinusoidal pattern. Additionally, PDMS surface structures can be generated using confined crumpling, which is triggered when applying a compressive force to a confined thin plate [28]. Crosby’s group, for instance, fabricated PDMS surface patterns through inflation and then deflation to compress an array of circular plates that were hexagonally arranged [28]. First, a PDMS substrate with an array of holes was clamped over a hole. The substrate was then inflated with air, which was followed by bonding a PDMS film over the substrate. After deflation, the resulting compressive stresses created a surface array of microstructures.

In this work, we applied the theory of circular plate buckling [29,30] to micromanufacturing, fabricating an array of curved PDMS film microstructures on PDMS substrate via mechanical stretching. Bidirectional mechanical strains were applied to a flat PDMS sheet with a square distributed hole array. Following that, a PDMS film was glued on the surface of the prestrained holes. Upon release of the prestrains, the buckling of circular films occurred, and the curved film microstructure arrays were formed spontaneously. The surface microstructures thus obtained are robust and uniform, possessing smooth profiles. They could serve as a microlens, through which optical imaging can be realized. The fabrication process was simulated to understand the mechanism of the circular film buckling induced by mechanical stretching. The simulation result coincides with the experimental result, which theoretically verifies the feasibility of creating the curved film microstructures through mechanical stretching. The fabricated microstructure arrays have a pitch of 350 μm. Compared to this method, higher resolution for microstructures could be achieved by employing other microfabrication techniques like micro-milling [31] and hot embossing [32]. Vecchione et al. reported microchannels with a depth of 3 μm and a width of 5 μm that were achieved through micro-milling [31], while Yun et al. fabricated dot patterns at intervals of 7–20 μm by using hot embossing [32]. However, our method could result in film microstructures that are difficult to obtain with other approaches. For example, micro-milling may not be suited to machining film microstructures made of soft materials. Hot embossing could be used for shaping soft materials; however, it generally could not lead to film microstructures with a sealed cavity. The surface patterning strategy presented in this paper provides a new idea of design and fabrication of the desired surface structure, which is very significant to many applications, such as optics and micro-electromechanical systems (MEMS).

## 2. Materials and Methods

The curved film microstructure arrays were fabricated as the following. We patterned a square arranged array of photoresist cylinders (20 × 20 in array configuration, 250 μm in diameter, 250 μm in height, and a distance of 350 μm between centers of two adjacent cylinders) onto a silicon wafer. Following that, Dow Corning Sylgard^®^ 184 PDMS precursor was mixed with curing agent (10 to 1 by weight). To ensure thorough mixing, the PDMS mixture was agitated with an ultrasonic oscillator for 5 min, which was followed by degassing under vacuum for 30 min. The degassed mixture was then cast onto the photoresist cylinder array and cured at room temperature for 36 h before a 1 mm thick PDMS sheet with hole array (250 μm in diameter) on the surface was yielded by mechanical peeling.

**Figure 1:**
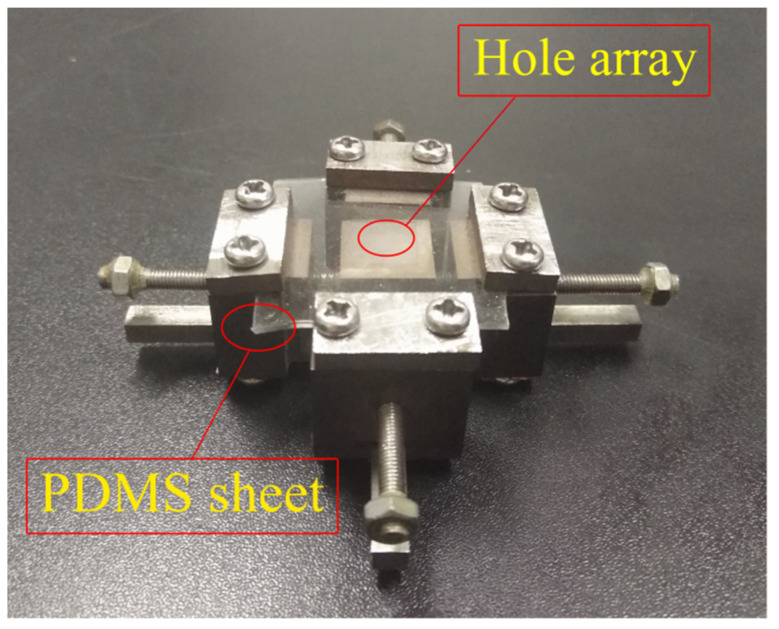
The tool used for stretching the sample.

**Figure 2:**
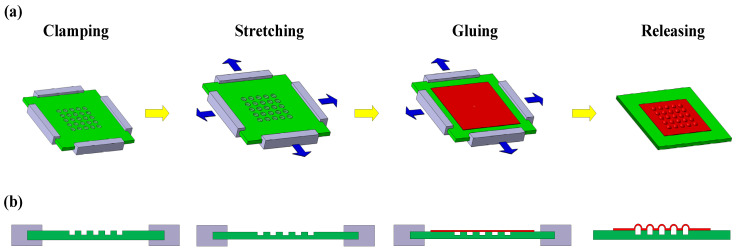
(**a**) Fabrication procedure of curved film microstructure array; (**b**) Forming process of film microstructures corresponding to fabrication steps.

A tool used for stretching the sample was designed and manufactured, which is thread-driven with a screw lead of 100 μm (Figure 1). The fabrication procedure of the curved film microstructure array is shown in Figure 2a. The PDMS sheet (30 mm × 30 mm × 1 mm) was placed on the sample stage of the stretching tool and clamped on four edges with the hole array in the central region as shown in Figure 1 (there is a distance of 11.55 mm between the borders of the hole array and the PDMS sheet for each of the square sides), and then stretched to 20% strain in two planar perpendicular directions simultaneously. The two stretching directions were parallel to the two directions of the hole arrangement, respectively. A BOPET film (biaxially-oriented polyethylene terephthalate) coated with a thin layer of uncured PDMS (around 4 μm in thickness) was placed on the surface of the strained holes of the PDMS sheet, and then removed. As a result, a layer of uncured PDMS was left on the surface of the strained holes. Immediately after that, a crosslinked PDMS film (18 μm in thickness), deposited on a BOPET film coated with a 20 μm thick film of cured unexposed SU-82005 photoresist (Microchem^®^, Newton, MA, USA), was laid on top of the array of strained holes so that the crosslinked PDMS film came into contact with the uncured PDMS layer. The sample was left to rest at the ambient temperature for 48 h to crosslink the uncured PDMS layer and bond the PDMS film to the array of strained holes.

The assembly was sprayed with SU-8 developer (Microchem^®^, Newton, MA, USA), which resulted in dissolution of the unexposed photoresist film that lay between the crosslinked PDMS film and the BOPET film, and thus removal of the BOPET film. The surface of the PDMS film was rinsed successively with fresh developer and deionized water before drying. Finally, the displacements applied to the PDMS sheet were released in both planar directions simultaneously, which created an array of curved film microstructures. The forming process of film microstructures corresponding to the fabrication steps is shown in Figure 2b.

The two-dimensional (2D) morphology of the curved film microstructures was assessed by using an optical microscope (Olympus STM6-F10-3, Olympus Co., Tokyo, Japan), while the 3D morphology of the curved film microstructures was assessed by using a laser scanning confocal microscope (Nikon A1+, gold-coated, Nikon, Tokyo, Japan). The 2D cross-sectional view of the curved film microstructures was examined by optical microscope (Nikon SMZ1270, colored film microstructures, Nikon, Tokyo, Japan). The 2D surface profile of a typical curved microstructure was characterized by profiler (VeecoDektak 150, Veeco, Plainview, NY, USA).

## 3. Results and Discussion

**Figure 3:**
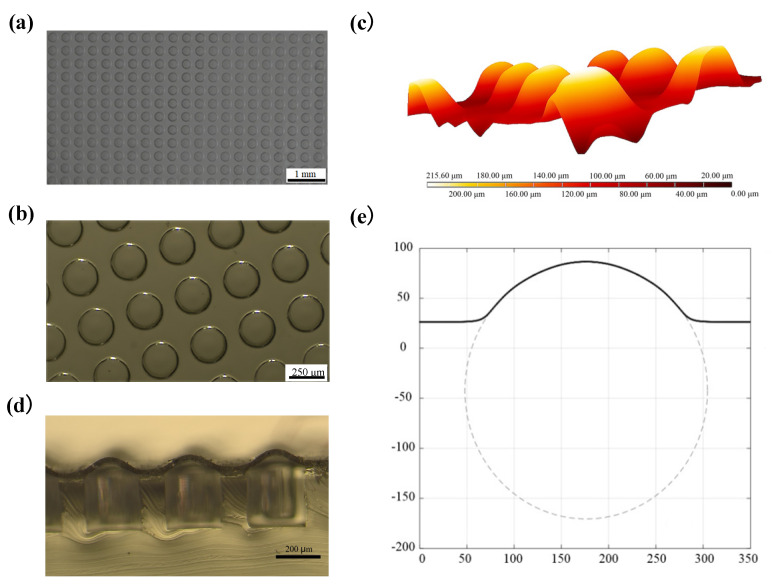
(**a**) Optical microscope image of the microstructures (magnification: 50×), illustrating the order and shape uniformity of the array structure; (**b**) Optical microscope image of the microstructures (magnification: 200×), displaying the two-dimensional (2D) morphology of the microstructures; (**c**) three-dimensional (3D) surface profiles of the fabricated film microstructures measured by using a laser scanning confocal microscope; (**d**) 2D cross-sectional view of the fabricated film microstructures; (**e**) The 2D surface profile of a typical curved film microstructure (solid line) and the fitted circle (dashed line).

Figure 3a,b display the 2D morphology of the fabricated film microstructure array. The 2D profiles appear very uniform, showing a circular shape with a diameter of around 250 μm, which is nearly equal to the diameter of the holes of the PDMS sheet. The 3D surface topography of the film microstructures is presented in Figure 3c, and the 2D cross-sectional view of the film microstructures is presented in Figure 3d. Besides good uniformity, the smooth connection with the flat film at the bottom of the microstructures is observed from the figures. The 2D surface profile of a typical curved film microstructure is displayed in Figure 3e, which also shows the fitted circle that has a radius of 128.4 μm. It can be seen in the figure that the 2D surface profile of the film microstructure has a circular arc shape with a height of about 60 μm.

**Figure 4:**
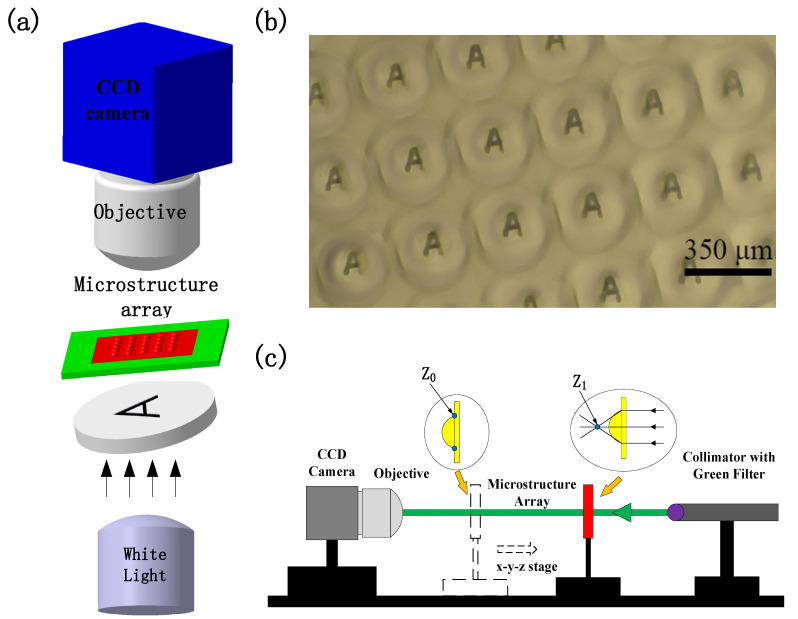
(**a**) Optical setup for demonstrating the lensing properties of the fabricated film microstructure array; (**b**) Optical microscope image of the multiple images of alphabet “A” through the fabricated film microstructure array; (**c**) The experimental setup for measuring the focal length of the curved film microstructure.

The fabricated film microstructure arrays can be employed as optical elements. A projection experiment was performed to illustrate the utility of these microstructures as microlens array for optical display application (Figure 4a). The film microstructure array was positioned on the sample stage of an optical microscope, and a printed transparency with an alphabet “A” (3 mm × 5 mm) on it was placed below the microstructure array. White light from the bottom illuminated the microstructure array through the printed transparency. Finally, an alphabet “A” was projected onto the focal plane of the microstructure array and imaged through the objective lens of the microscope. As Figure 4b shows, we observe a square array of the letter “A” on the microstructure array.

The focal length of the film microstructure array was measured using the experimental set up schematized in Figure 4c. A collimated light at a wavelength of 532 nm from a laser illuminated from the bottom of the microstructure array that was mounted on an *x-y-z* translation stage. First, a microscope was focused on the base surfaces surrounding the microstructures (Z_0_), which was used as the reference point. The stage was then moved further away from the microscope objective along the optical axis to the focal point (Z_1_) by finding the minimum laser spot in the microscope. The distance the stage was moved from the reference point Z_0_ to the focal point Z_1_ was the focal length of the microstructures. Since the microstructure profile was uniform over the microstructure array, the focal length of a single microstructure was obtained over the entire array. The focal length was measured to be approximately 1010 μm.

**Figure 5:**
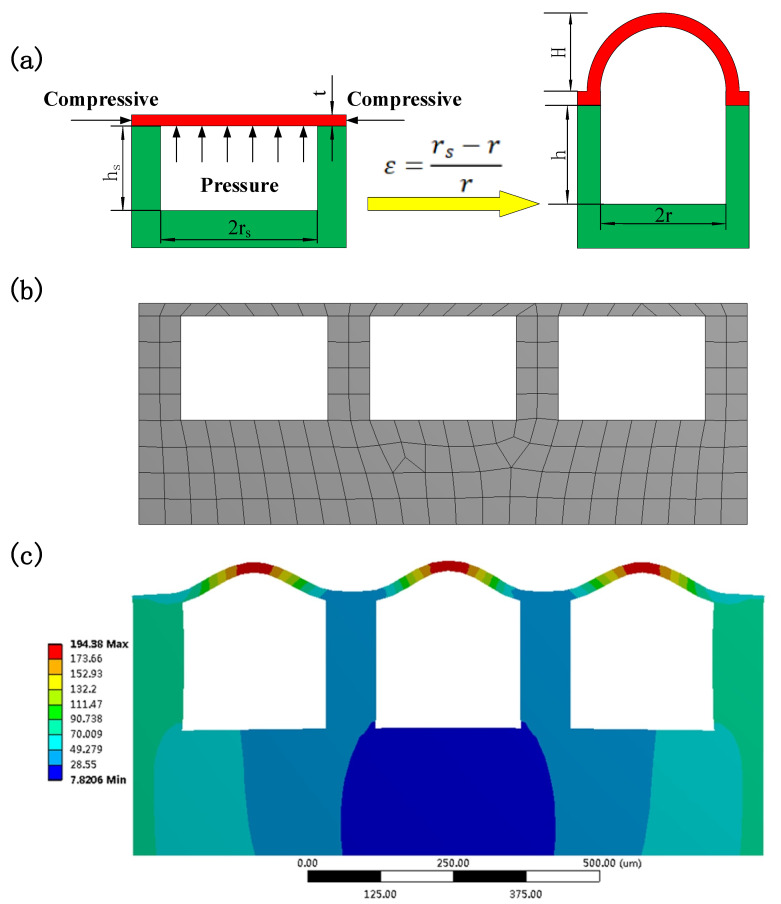
(**a**) Forces applied to an individual circular film when the prestrain is released (**left**), as well as the resulting film microstructure (**right**); (**b**) Finite element mesh used for numerical simulations; (**c**) Curved film microstructures obtained by using finite element simulation with the end face displacement and the pressure applied to the film. The color represents displacement (μm).

The curved film microstructures were formed through confined buckling of circular films induced by mechanical stretching that was carried out in the plane bidirectionally. The PDMS film was glued to the surface of the bidirectionally prestretched PDMS sheet with a square arranged hole array. Releasing the prestrains caused an equi-biaxial compressive stress to be generated at the edges of the circular films (Figure 5a). When the compressive stress exceeded a critical stress, the film buckling occurred, thus creating the curved film microstructure array. This critical stress for buckling is given by:

(1) $$\sigma_{c}=\frac{{}^{}k^{2}Et^{2}}{12(1-\gamma^{2})r_{s}{}^{2}}$$

where *k* is a numerical constant for buckling mode, *E* is the film elastic modulus, *t* is the film thickness, *γ* is Poisson’s ratio of the film, and *r_s_* is the initial radius of circular film (i.e., the radius of the strained holes of the prestretched PDMS sheet) [29,30]. 

As the film microstructure under study is formed, its equilibrium shape is selected via the competition between bending and stretching energies [33]. The stretching energy scales linearly with the film thickness *t*, resulting from in-plane strain. The bending energy scales as *t^3^*, vanishing only in flat configurations. When *t* is very small, the stretching energy prevails over the bending energy. In this case, the film tends to bend to reduce its in-plane strain, that is, the stretching energy. As a consequence, the circular films buckle under the action of the equi-biaxial compressive stress to create the curved film microstructures (Figure 5a).

In order to further study the mechanism of the formation of the curved film microstructure array, the numerical simulations are performed using a commercial finite element method (FEM) software ANSYS^®^ (ANSYS, Inc., Canonsburg, PA, USA). The calculation is carried out on an assembly, i.e., a PDMS film (18 μm in thickness) that is glued on a prestretched PDMS sheet (1150 × 500 μm) with hole array on its surface. The hole array has the same geometry as those fabricated in the experiments. A bi-dimensional finite-element mesh is used and is shown in Figure 5b. For the material properties, the PDMS is considered as linear elastic isotropic material because there is no large displacement during the fabrication process of the curved film microstructure array. Based on the real experimental situation, the boundary conditions are chosen as follows: the bottom surface of the PDMS film is bonded to the surface of the strained holes of the prestretched PDMS sheet; there is no displacement of the end faces of the PDMS film in the direction perpendicular to the film surface. The prestrain applied to the PDMS sheet is 20%, and it is uniform over the surface holes of the PDMS sheet as observed via optical microscopy. Accordingly, the diameter of the strained holes, as measured by optical microscopy, is about 300 μm. When the prestrain is released, there is a displacement of the end faces of the assembly in the opposite direction to the prestretching. As a result, the dimensions of the holes change back to the original ones (250 μm in diameter and 250 μm in height), which was observed via optical microscopy in the experiment. The simulation result shows that the curved film microstructure array is generated upon release of the prestrain. Assuming the elastic modulus *E* = 3.99 MPa and Poisson’s ratio *γ* = 0.49 for PDMS, the height of the film microstructures is calculated to be 29.8 μm.

Additionally, the change in the volume of the holes that are below the PDMS film could play a role in the generation of the curved film microstructure array. The mechanical stretching of the PDMS sheet is simulated with 20% strain, which indicates an increased volume of the holes. Therefore, besides the compressive stress that is generated at the edges of the circular films, a pressure *P* from the bottom is applied to the PDMS circular films above the holes as the prestrain is released (Figure 5a). Since the dimensions of the strained holes change back to the ones before prestretching, the pressure can be given by *P* = *P*_atm_ ((*r_s_^2^/r^2^*) (*h_s_/h*) − 1), where *P*_atm_ is the atmospheric pressure, *r_s_* and *r* are the radius of the holes after and before prestretching, and *h_s_* and *h* are the height of the holes after and before prestretching. When the numerical analysis is carried out with the end face displacement and the pressure applied to the film, it can still be seen that the curved film microstructure array is created (Figure 5c). In this case, the height of the microstructures is found to be 75.6 μm, which is larger than that of the microstructures obtained with only end face displacement, and relatively close to the measured value 60 μm. As shown in the figure, the achieved microstructures appear very uniform, and they closely resemble the fabricated ones in shape. Hence, the simulation results theoretically verify the feasibility of creating the curved film microstructure by mechanical stretching.

**Figure 6:**
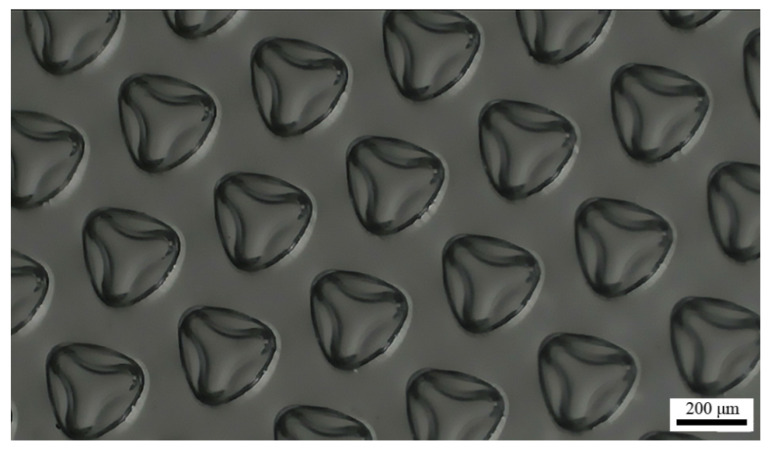
Optical microscope image of the microstructures fabricated through applying 60% prestrain.

The quantity *k* used in Equation (1) denotes the way that PDMS film buckles. There is actually more than one way that the film buckles. In other words, more than one type of microstructure could be formed through film buckling. The applied prestrain has a great effect on the shape of the fabricated microstructures, which was observed in the experiments. For instance, when 60% prestrain was applied to the PDMS sheet during the fabrication, the resultant microstructures have three lobes rather than a spherical surface (Figure 6). Since the spacing of the fabricated microstructures was large enough, no cross-talk between adjacent microstructures was observed. Therefore, the cross-sectional area of the strained hole should be equal to the surface area of the spherical film microstructure. Based on this conservation of surface area, the relationship between the film microstructure geometry and the applied prestrain can be described by:

(2) $$\frac{H}{r}=\sqrt{\varepsilon (2+\varepsilon )}$$

where *H* is the height of the film microstructure, and *ε* is the applied prestrain [28]. It is shown in Equation (2) that the height of the film microstructure increases with increased prestrain. If *ε* is relatively small, the film microstructure is formed with a spherical surface of constant curvature, which can resist the applied bending moment. When a large prestrain is applied during the fabrication process, the film buckling will create the bifurcated film microstructures such as those shown in Figure 6. The bifurcation will further decrease in-plane strain to resist the increased bending moment [34]. In addition, this approach should be scalable to much smaller length scales [28], which implies that higher resolution for the film microstructures might be achieved. The geometry of the microstructures depends on the circular films’ initial geometry and the applied force during fabrication [34]. Therefore, tuning the parameters related to these factors should be an effective strategy to increase the resolution.

## 4. Conclusions

We have demonstrated a simple and flexible method to create curved film microstructure arrays, which is based on confined buckling of circular films induced by mechanical stretching. The fabricated microstructures were very uniform, and could be used as microlens arrays with a focal length of 1010 μm. The patterning mechanism was studied through theoretical analysis and numerical modelling. The simulation results match well with the experimental results. Compared with other ways, mechanical stretching is a relatively simple way to induce the film buckling. Furthermore, the shape of the resulting film microstructures could be easily controlled by using this patterning technique because the prestrain applied during the fabrication could be easily controlled via mechanical stretching. This patterning method offers new perspective in the design and fabrication of desired surface microstructures, which is of great significance to a variety of applications such as optical microdevices, microfluidic devices, tuning of friction or adhesion, and so on. The future work is to investigate the resolution for microstructures that can be achieved with this microfabrication technique.
